# Adaptation of the personal social capital brief scale for the measurement of the offline and online social capital in Italy

**DOI:** 10.1371/journal.pone.0272454

**Published:** 2022-09-01

**Authors:** Elisa Menardo, Roberto Cubelli, Giulia Balboni

**Affiliations:** 1 Department of Human Sciences, University of Verona, Verona, Italy; 2 Department of Psychology and Cognitive Sciences, University of Trento, Rovereto, Italy; 3 Department of Philosophy, Social Sciences and Education, University of Perugia, Perugia, Italy; University of Bologna, ITALY

## Abstract

Social Capital refers to the resources associated with durable and trustworthy social connections. Social Capital can be developed through offline and online relationships. It can be distinguished between cognitive Social Capital (perception of trustworthiness, reciprocity, and support) and structural Social Capital (density of social networks and membership, and participation in groups and associations). It can also be distinguished between bonding Social Capital (resources associated with informal networks; i.e., neighbors, friends, colleagues) and bridging Social Capital (resources associated with formal networks; i.e., community service, cultural, religious or political groups/associations). The different forms and dimensions of Social Capital may have distinct effects on health outcomes and self-rated health. Therefore, public health researchers need valid and reliable instruments to investigate Social Capital. However, valid instruments including the measurement of online Social Capital are not available. The Personal Social Capital Scale aims to assess bonding and bridging Social Capital by means of cognitive and structural items. In the present investigation, three studies were carried out (*N* = 1149) to adapt the Personal Social Capital Scale to develop the Personal On-Offline Social Capital Brief Scale, a brief scale for measuring online and offline bonding and bridging Social Capital in Italy. Factorial structure and convergent/divergent validity in relation to scales measuring constructs with different patterns of relationships with bonding and bridging Social Capital (i.e., social support and stress; sense of community and health) were also investigated. Overall, these studies provide evidence of reliability and validity related to the internal structure of the Personal On-Offline Social Capital Brief Scale in measuring online and offline bonding and bridging Social Capital and discriminating them from similar constructs. This scale is a useful instrument for planning public health interventions.

## Introduction

The notion of Social Capital refers to the resources associated with durable and trustworthy social network connections [1, 2]. It was introduced over a hundred years ago [3], and several classifications have been proposed over the years [4, 5].

A first distinction concerns the level at which the Social Capital is measured. Personal Social Capital regards personal resources, whereas community Social Capital concerns collective resources shared between members of one community [4, 6]. A second distinction is between cognitive Social Capital, which is about the perception of trustworthiness, reciprocity, and support, and structural Social Capital, which regards the density of social networks and membership and participation in groups and associations [5, 7–9]. A third important distinction is between bonding and bridging Social Capital [5, 10–12]. Bonding Social Capital is about resources linked to connections “within groups” (i.e., family members, neighbors, friends, colleagues). Bridging Social Capital includes resources linked to connections “across groups” (i.e., formal social networks).

Finally, Social Capital can be developed through relationships in real life and virtual environment. Social network sites (e.g., Facebook, Instagram, Twitter) are social environments where people can build Social Capital by creating new connections (e.g., meeting new friends) and strengthening or maintaining offline relationships [13, 14]. Online relationships are potential sources of social support [15]. At the same time, they promote relationships in real life [16, 17]. However, even if social network sites increase Social Capital [13], only a few studies have considered “online friends” as a source of Social Capital (e.g., [18].

Studies found different relationships between Social Capital and health outcomes [5, 10, 19] and self-rated health [4, 5, 20–22]. Different dimensions of Social Capital may account for this “double-edged phenomenon” [4]. Indeed, only personal Social Capital is associated with higher self-reported health [21, 23] and lower risks of depression [24]. In contrast, only community Social Capital may be relevant for preventing suicide in the elderly [25]. Whereas social trust (cognitive Social Capital) was found to be related to self-rated health, social participation [21] and group membership [26] (structural Social Capital) had null or negative effects on health [26–29]. Additionally, preventive health care practices could be facilitated by high levels of bridging Social Capital and, at the same time, hindered by high levels of bonding Social Capital [30]. Self-rated health was associated with bridging Social Capital in cross-sectional studies [10, 12] and bonding Social Capital in longitudinal research [31]. Finally, online relationships may enhance positive health outcomes [32, 33], especially when offline Social Capital is scarce [34], and may promote relationships in real life [16, 17] that, in turn, improve health and wellbeing [26]. However, negative health-related outcomes are linked to excessive and compulsive use of information and communication technologies (ICT) [35] or connection overload [36].

Several measurement tools of Social Capital are available (for a review, see [8, 9, 37]). Nonetheless, some of them (e.g., Position Generator or Resource Generator, [38]) are based on the listing approach, which is problematic for data collection and scoring. A recent tool, the Adapted Social Capital Assessment Tool (A-SCAT) [39], is based on the modern psychometric approach, and it was developed for multi-country large-scale studies. Yet, it does not distinguish Social Capital from its function (e.g., provide support) [37]. To overcome this limitation, and focus on what Social Capital *is*, the Personal Social Capital Scale (PSCS) was developed [11].

The PSCS aims to assess bonding and bridging Social Capital at the individual level [11] by means of cognitive (i.e., perception of trustworthiness, reciprocity, and support) and structural (i.e., size of networks and frequency of connections) items. The PSCS is composed of 10 items (42 subitems) based on a 5-point Likert scale and showed excellent validity and reliability in China [11] and South America [40]. Additionally, two brief versions of the PSCS (16 and 8 single items) have been developed [39]. They showed good psychometric proprieties in the Asiatic context [37].

To our knowledge, the PSCS is the only Social Capital measurement tool that allows investigating the cognitive and structural aspects of personal bonding and bridging Social Capital [8]. However, it was developed and validated only in Asiatic and South American contexts, and it does not allow the measurement of online Social Capital.

For this reason, the present investigation aimed to adapt the PSCS to develop the Personal On-Offline Social Capital Brief Scale, a tool for measuring both the offline and online Social Capital in Italy. In the first study, we developed a brief version of the Italian PSCS (PSCS-Brief Italian Version). First, we selected the most informative items of the Italian version of the PSCS ([41]; see the following for the use of this scale: [42–45]), in accord with the methodology proposed by Wang et al. [37]. Then, we ran a confirmatory factor analysis (CFA) to investigate the factorial structure of the selected items. In the second study, we developed the Personal On-Offline Social Capital Brief Scale by introducing items for the measurement of online Social Capital, and verified its factorial structure and reliability. Finally, in the third study, we investigated the convergent/divergent validity of the Personal On-Offline Social Capital Brief Scale in relation to scales measuring constructs with different patterns of relationships with bonding and bridging Social Capital (i.e., social support and stress; sense of community and health). This study is particularly relevant for identifying distinct dimensions of online and offline SC that can be used for planning public health interventions.

## First study: Development of the personal social capital scale-brief Italian version

This study aimed to develop the PSCS-Brief Italian Version selecting the most informative items from the PSCS-Italian Version and verifying its factorial structure via CFA.

### Materials and methods

#### Instruments

*Personal social capital scale-Italian version*. The Italian version of the PSCS [41] comprises 10 composite items with a total of 54 subitems, each rated on a 5-point Likert scale (A few/None-A lot/All). Examples of contents investigated in the subitems are in Table 1. The first five composite items evaluated the individual’s involvement with informal networks (bonding Social Capital). The first four composite items assessed: (1) the network size, (2) the contacts with network members, (3) the trust in the network members, and (4) the support received upon request from the following informal networks, each of which was a subitem: (a) family members, (b) relatives, (c) people in their own neighborhood, (d) friends, (e) work colleagues/fellow students, and (f) fellow countrymen/region men or childhood friends/old classmates. The fifth composite item asked for the estimation of the number of members of all informal networks having the following resources, each of which was a subitem: (a) political power, (b) financial wellbeing, (c) broad connections with others, (d) high reputation/influence, (e) at least a high school educational level, and (f) professional competence. The remaining five composite items assessed involvement with formal networks (bridging Social Capital). In the first four composite items, the individuals were asked to estimate (1) the number of associations/groups present in their own community, (2) participation in activities, (3) the representation of their own rights and interests, and (4) the support received upon request from the following types of associations/groups, each of which was a subitem: (a) economic and professional, (b) community service, (c) religious or political, (d) cultural, and (e) recreational associations/groups. The last composite item asked about the number of associations/groups having the following resources, each of which was a subitem: (a) noteworthy decision-making power, (b) solid financial bases, (c) broad social connections, and (d) extensive social influence.

**Table 1:** Subitem-total correlation coefficients and Cronbach’s alpha if subitem deleted for the 16 subitems organized in eight composite items of the personal social capital scale-brief Italian version.

Composite Item	Content Investigated in the Subitem	Subitem-total correlation coefficient	Cronbach’s alpha if subitem deleted
BO1	1. Number of the individual’s friends	.405	.897
BO1	2. Number of the individual’s work colleagues/fellow students	.295	.898
BO2	3. Number of the individual’s friends they can trust	.399	.897
BO2	4. Number of the individual’s work colleagues/fellow students they can trust	.392	.897
BO3	5. Number of the individual’s friends who will definitely help them upon their request	.459	.896
BO3	6. Number of people in the individual’s neighborhood who will definitely help them upon their request	.466	.896
BO4	7. Number of the individual’s friends, work colleagues/fellow students, people in the neighborhood, family members, relatives, fellow countrymen/region men or childhood friends/old classmates who have broad connections with others	.460	.896
BO4	8. Number of the individual’s friends, work colleagues/fellow students, people in the neighborhood, family members, relatives, fellow countrymen/region men or childhood friends/old classmates who have a high reputation/influential	.418	.897
BR1	9. Number of community service associations/groups in the individual’s community	.497	.895
BR1	10. Number of cultural associations/groups in the individual’s community	.453	.896
BR2	11. Number of community service associations/groups in the individual’s community that represent their rights and interests	.556	.895
BR2	12. Number of cultural associations/groups in the individual’s community that represent their rights and interests	.471	.896
BR3	13. Number of community service associations/groups in the individual’s community that will support them upon their request	.552	.895
BR3	14. Number of religious or political associations/groups in the individual’s community that will support them upon their request	.497	.896
BR4	15. Number of community service, cultural, religious, political, recreational, leisure, and economic and professional associations/groups in the individual’s community that possess broad social connections	.533	.895
BR4	16. Number of community service, cultural, religious, political, recreational, leisure, and economic and professional associations/groups in the individual’s community that possess extensive social influence	.511	.895

*Note*. BO = Bonding Social Capital; BR = Bridging Social Capital.

The Italian version of the PSCS was adapted from the original English version in agreement with the ITC Guidelines on Translation and Adapting Tests [46]. Two Italian professors of psychology with expertise in scale development and Social Capital translated the items into Italian. Then, a professional Italian-English interpreter verified the translation. Finally, one psychologist and one sociologist independently evaluated each item. A first field test was conducted with 19 adults to investigate whether the items and instructions were clear, concise, and exhaustive and if the answer scale was applicable for each item. Based on their answers, we decided to modify the original English version of the PSCS composite items on bridging Social Capital that grouped all associations into two groups (governmental, political, economic, and social associations/groups; cultural, recreational and leisure associations/groups) into one subitem for each of the following associations: economic and professional, community service, political or religious, cultural, and recreational. A second field test was performed with 12 other adults.

*Balanced Inventory of Desirable Responding-6 (BIDR-6) short form*. The 16-item scale assesses the unconscious tendency toward socially desirable responses [47, 48], and it was used to detect social desirability responding (i.e., the systematic tendency to give overly positive answers that make the respondent look good). Individuals were considered simulators when their total score exceeded the 95^th^ centile obtained by the normative group [47]. This scale has shown good psychometric proprieties (internal consistency and factor structure) [47].

#### Participants

The participants were 385 adults (45% men; mean age = 45.3 years [*SD* = 7.1], range = 28–68 years) living in towns located in Northern and Central Italy with populations fewer than 5 thousand individuals (38%); 5–50 thousand individuals (37%); 50–250 thousand individuals (15%); and more than 250 thousand individuals (10%). Among the participants, 28% had a third-year secondary school or lower education level, 45% had a secondary school education level, and 27% had a postsecondary school or a higher education level. Most of the participants (88%) reported being employed, while a few reported being unemployed (9%) or retired (3%). Participants had no kinship ties.

The participants were selected among the participants of the previous two studies on the relationships between sociocultural level and adaptive behavior and between sociocultural level and personality [43, 45]. Among the 454 participants who had agreed to take part in the study, 69 (18%) were excluded: 26 did not complete all the administered questionnaires, and 43 exceeded the cutoff score at the BIDR-6 of social desirability responding.

#### Procedure

All participants filled out a paper and pencil copy of the PSCS Italian Version and then of the BIDR-6 (approximately 5 minutes each). Data collection took place between June 2011 and November 2013. For a detailed description of the procedure, see [43, 45].

This study was performed in accordance with the ethical standards laid down in the 2008 Seoul version of the Declaration of Helsinki. Written informed consent was obtained from each participant, and they did not receive any form of incentive. The Institutional Review Board Comitato Etico Pediatrico Regione Toscana, Azienda Ospedaliera Universitaria Meyer, Firenze, Italia, approved the the developed PSCS Italian Version (#9/2016).

#### Data analysis

*Selection of the most informative items*. We followed the multistep approach employed by Wang and colleagues [37]. Two of the original 10 composite items that assessed contacts within each informal network (bonding Social Capital) and participation in the activities of each association/group (bridging Social Capital) were deleted because they evaluated investment in Social Capital rather than Social Capital itself. Then, for each of the remaining eight composite items, we selected the two more informative subitems with the highest subitem-total correlation coefficient and with the lower corrected Cronbach’s alpha coefficient (i.e., Cronbach’s alpha of the scale if the subitem was deleted). Thus, eight composite items, four each for bonding and bridging Social Capital, composed of two subitems each, were selected to develop the PSCS-Brief Italian Version.

*Confirmatory factor analysis*. We checked for normal distribution of each PSCS-Brief Italian Version subitem and for the presence of univariate outliers for the PSCS-Brief Italian Version total score (± 3.29 standard deviation from the group mean). Then, we normalized the subitem score distributions. Following Wang et al.’s [37] approach, we computed the mean of the scores on the two subitems of each of the eight composite items. For each of these mean scores, we checked for normal distribution and presence of univariate outliers and of multivariate outliers using Mahalanobis distance. Every time we excluded participants, we normalized each subitem score distribution, and we recomputed mean scores for the two subitems of the same composite item. These eight composite item scores, four for each bonding and bridging Social Capital, were then used for the CFA.

The R package lavaan [49] was used to investigate the goodness of fit of the 2-factor model composed of the two bonding and bridging Social Capital latent factors loaded by the corresponding four two-subitem mean scores. The metric of the two latent variables was assigned by fixing the factor loading of the first two-subitem mean scores of each factor as equal to 1. In addition to the Satorra-Bentler scaled Chi-square statistic (S-Bχ^2^), comparative and residual-based fit indices were used: the robust comparative fit index (rCFI), the Robust Root-Mean-Square Error of Approximation (rRMSEA) with associated 95% confidence intervals (CIs), and the standardized root-mean-square residual (SRMR) [50–52]. As indicated in the literature (e.g., [50]), reasonable fit is suggested by values close to .95 for rCFI, smaller than .05 for rRMSEA, and smaller than .08 for SRMR.

CFA results could be significantly influenced by a few powerful cases (i.e., individuals impacting model results) [53]. For this reason, the sensitivity analysis of cases was performed using the R package faoutlier [54]. Following the approach proposed by Pek and Callum [53], factor model-based influential cases were identified by an excessive likelihood distance (i.e., excessive influence of an individual case on the overall model fit). The benefits of investigating influential cases are widely recognized [53] as it helps to avoid drawing conclusions driven by few cases [55]. As suggested by Aguinis and colleagues [56], we reported the results obtained with and without influential cases.

We also checked whether the 2-factor model performed better than the theoretically plausible 1-factor model obtained by collapsing the two latent factors into one latent factor. For this aim, we used ΔS-Bχ^2^ and ΔrCFI as fit indices. A significant ΔS-Bχ^2^ and a ΔrCFI (which is less affected by sample size) value > 0.01 are required [57]. Finally, Akaike’s Information Criterion (AIC) was generated, with a lower value indicating a better fitting model [52].

The appropriateness of the CFA sample size was verified by calculating the statistical power of the model [58] using the R package “semPower” [59].

### Results

#### Selection of the most informative items

The first three original PSCS Italian version composite items of the bonding Social Capital assessed were: (1) the network size, (2) the individual’s trust in the network, and (3) the support received from each of the following informal networks, each of which was a subitem: (a) family members, (b) relatives, (c) people in the own neighborhood, (d) friends, (e) work colleagues/fellow students, and (f) fellow countrymen/region men or childhood friends/old classmates. In agreement with the described selection procedure, we identified the two subitems with the highest subitem-total correlation coefficient and the lowest alpha if the subitem was deleted for each of these composite items. For all the composite items, these two subitems were those concerning friends and fellow countrymen/region men or childhood friends/old classmates. However, there could be overlap between these two member networks. Therefore, in agreement with Wang and colleagues [37], to avoid redundancy, for each composite item, we kept the subitem concerning friends. In contrast, we substituted the subitem on fellow countrymen/region men or childhood friends/old classmates with the subitem among the remaining items with the highest item-total correlation coefficient and the lowest corrected Cronbach’s alpha. In this way, as seen in Table 1, as a second subitem, we selected work colleagues/fellow students for the two composite items on network size and individual trust and people in their own neighborhood for the composite item on support received.

The fourth composite item of bonding Social Capital asked the number of members for all networks having the following resources, each of which was a subitem: (a) political power, (b) financial wellbeing, (c) broad connections with others, (d) high reputation/influence, (e) at least high school educational level, and (f) professional competence. Based on the highest subitem-total correlation coefficient and the alpha if subitem deleted, the subitems selected were those regarding broad connections with others and high reputation/influential resources.

Regarding bridging Social Capital, the first three original PSCS Italian version composite items assessed: (1) the number of associations/groups present in the community, (2) the representation of the individual’s own rights and interests, and (3) the support received upon request from the following five associations/groups, each of which was a subitem: (a) economic and professional, (b) community service, (c) religious or political, (d) cultural, and (e) recreational associations/groups. As seen in Table 1, based on the highest subitem-total correlation coefficient and alpha if the subitem was deleted for each of these composite items, the subitem regarding community service associations/groups was always selected as the first subitem. A second subitem was selected on cultural associations/groups for the composite item regarding the number of associations/groups present in the community and the representation of the individual’s own rights and interests and on religious or political groups/organizations for the composite item regarding the support received from the network upon request.

The last composite item of bridging Social Capital asked the number of associations/groups having the following resources, each of which was a subitem: (a) noteworthy decision-making power, (b) solid financial bases, (c) broad social connections, and (d) extensive social influence. Based on the highest subitem-total correlation coefficient and the alpha if subitem deleted, the subitems selected were those regarding broad social connections and extensive social influence resources.

In this way, the eight composite items of the PSCS-Brief Italian Version, each composed of two subitems, were selected. The subitem-total correlation coefficients ranged from .295 to .556 (mean = .460; median = .463), and alpha if item deleted ranged from .895 to .898 (mean, median = .896).

#### Confirmatory factor analysis

No univariate outliers were found for the PSCS-Brief Italian Version total score, while one multivariate outlier was found and excluded for the eight composite item scores (*n* = 384). The composite item score distributions were still far from a multivariate normal distribution (based on Mardia’s test), so the maximum likelihood estimator with mean-adjusted chi-square test (MLM estimator), which is robust to nonnormal score distributions, was used for the CFA.

The 2-factor model did not meet the criteria for well fit (S-Bχ^*2*^_(19)_ = 91.3, *p* < .001; rCFI = .871; rRMSEA = .106, 90% C.I. = .085, .129; SRMR = .063; AIC = 6672). We detected and excluded 6 influential cases (2%) that worsened the model fit (i.e., likelihood distance < - 2.5) (no differences were found between them and the remaining 378 participants in the subitem scores, age, gender, educational level, population size of the town, and occupational status). We normalized the subitem scores and computed the mean of the two-subitem scores of each composite item. The obtained 2-factor model had a better fit but still was not well enough (S-Bχ^*2*^_(19)_ = 73.9, *p* < .001; rCFI = .905; rRMSEA = .092, 90% C.I. = .070, .114; SRMR = .055; AIC = 6528), and the total explained variance was quite low (equal to 19%).

Following the modification indices, we ran a second model releasing the correlation coefficient between the residual of the scores of the two composite items of bonding Social Capital regarding the number of friends and work colleagues/fellow students and the support received from friends and people in the neighborhood. The obtained 2-factor model had an adequate fit (S-Bχ^*2*^_(18)_ = 54.9, *p* < .001; rCFI = .937; rRMSEA = .077, 90% C.I. = .054, .101; SRMR = .052; AIC = 6509) and slightly higher total explained variance (equal to 20%). Moreover, the obtained 2-factor model was better than the 1-factor model, with the two bonding and bridging Social Capital dimensions collapsing in one dimension (S-Bχ^2^_(20)_ = 157.4, *p* < .001; rCFI = .763; rRMSEA = .144, 90% C.I. = .121, .162; SRMR = .077; AIC = 6618). Indeed, ΔS-Bχ^2^ was statistically significant (ΔS-Bχ^2^_(2)_ = 102.5, *p* < .001), ΔrCFI was higher than .01 (ΔrCFI = .174), and the AIC was lower for the 2-factor model.

**Fig 1:**
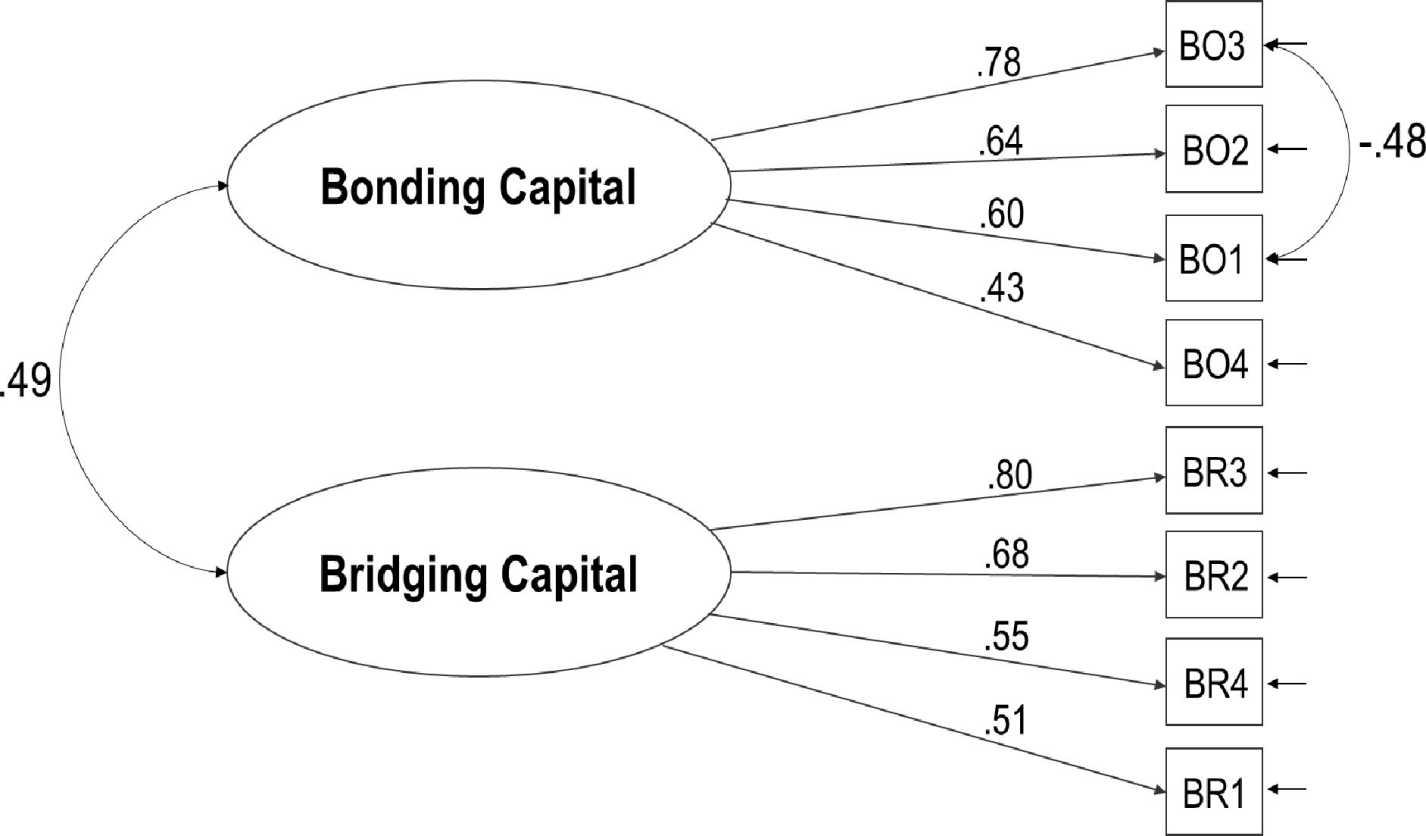
Confirmatory factor model of the 2-factor model of the personal social capital scale-brief Italian version.

As shown in Fig 1, the factor loadings ranged from .43 to .80 (mean, median = .62) and were all statistically significant. The interfactor correlation was moderate. The Cronbach’s alpha was equal to .76 (McDonald’s omega = .81) for the eight composite items and equal to .66 and .72 for the bonding and bridging four composite items, respectively. The statistical power of the model was excellent (.99).

### Discussion

In the first study, we developed the PSCS-Brief Italian Version by selecting the 16 most informative subitems from the PSCS-Italian Version. CFA showed that these subitems were organized into eight composite items consisting of two subitems each, four for each of the two interrelated dimensions of bonding and bridging Social Capital. The selected subitems investigated resources that are associated with informal networks of friends, colleagues/fellow students, and neighbors (bonding Social Capital), and with community service, cultural, and religious or political associations/groups (bridging Social Capital).

The rejected subitems concerned resources associated with family members, relatives, and fellow citizens or old childhood friends/old classmates (bonding Social Capital),) and recreational and economic/professional associations/groups (bridging Social Capital).

These results are quite consistent with those obtained with the Chinese population [37]. For both Italian and Chinese populations, the most relevant informal networks are friends and colleagues/fellow students, and the most relevant resources for informal and formal networks are broad connections and social influence. In other words, allowing individuals to access and strengthen their own networks seems to be a valuable feature of Social Capital.

To select the most informative items of the PSCS-Brief Italian Version, we replicated the approach used by the authors of the original PSCS based on the subitem-total correlation coefficient and the alpha if the subitem was deleted. However, alternative approaches are available. For example, the information function based on the Item Response Theory describes how well and precisely each item measures the construct. Therefore, further investigations are necessary to confirm the present results.

Additionally, the total variance explained by the 2-factor model of the PSCS-Brief Italian Version was low. Therefore, we developed a new version of the scale by adding items assessing online Social Capital to overcome this limit.

## Second study: Development of the personal on-offline social capital brief scale

The second study aimed to develop the Personal On-Offline Social Capital Brief Scale and verify its factorial structure.

### Material and methods

#### Instruments

*Personal on-offline social capital brief scale*. Online contacts refer to people met online and with whom interactions are only online. For the bonding Social Capital dimension of the PSCS-Brief Italian Version, online contacts were introduced as the third informal network for each composite item and in the composite item concerning the assessment of the number of members with specific resources. For the bridging Social Capital dimension, the cultural associations/groups also included associations/groups having only online activities. In this way, the Personal On-Offline Social Capital Brief Scale was composed of 19 subitems organized into eight composite items of three or two subitems each, four for each bonding and bridging Social Capital dimension. Field tests were realized to investigate if the new items were clear, concise, and exhaustive and if the answer scale was applicable.

*BIDR-6 short form*. See the first study.

#### Participants

The participants were 283 adults (39% men, mean age = 40.5 years [*SD* = 14.7], range = 19–80 years) living in Perugia, a medium-sized town located in the center of Italy (160 thousand inhabitants). Most of them were employed (72%) and had at least thirteen years of education (77%) and either a high school (39%) or a university (38%) degree.

All participants were randomly selected among adults of a study on the relationships between SES, Cultural Capital, and Social Capital [60]) who had completed the Personal On-Offline Social Capital Brief Scale. Among the 304 selected participants, 21 (7%) were excluded because they exceeded the cutoff score of the BIDR-6 of social desirability responding.

#### Procedure

All participants completed an online version of the Personal On-Offline Social Capital Brief Scale followed by the BIDR-6 (approximately 5 minutes each). Data collection took place between October 2017 and November 2019. For a detailed description of the procedure, see [60].

This study was performed in accordance with the ethical standards laid down in the 2013 Fortaleza version of the Declaration of Helsinki. Written informed consent was obtained from each participant, and they did not receive any form of incentive. The Institutional Review Board Comitato Universitario di Bioetica, University of Perugia, approved the original study [60] (#2018-03R).

#### Data analysis

See the first study, confirmatory factor analysis.

### Results

No univariate outliers were found for the Personal On-Offline Social Capital Brief Scale total score; in contrast, one multivariate outlier was found for the eight composite item scores and excluded (*n* = 282). The MLM estimator was used given that the eight composite item score distributions were not multivariate normal distributions (based on Mardia’s test).

The 2-factor model did not meet the criteria for a good fit (S-Bχ^*2*^_(19)_ = 88.4, *p* < .001; rCFI = .864; rRMSEA = .120, 90% C.I. = .095, .146; SRMR = .066). We detected and excluded 12 influential cases (4%) that worsened the fit model (i.e., likelihood distance < - 3) (no differences were found between them and the remaining 270 participants in subitem score, age, gender, educational level, and occupational status). The obtained 2-factor model had a good fit (S-Bχ^*2*^_(19)_ = 54.5, *p* < .001; rCFI = .929; rRMSEA = .087, 90% C.I. = .061, .115; SRMR = .052; AIC = 4524), and the percentage of total explained variance was moderate (equal to 43%).

Moreover, the obtained 2-factor model was better than the 1-factor model, with the two bonding and bridging Social Capital dimensions collapsing into one dimension (S-Bχ^*2*^_(20)_ = 79.7, *p* < .001; rCFI = .823; rRMSEA = .133, 90% C.I. = .103, .164; SRMR = .080; AIC = 8244). Indeed, ΔS-Bχ^2^ was statistically significant (ΔS-Bχ^2^_(1)_ = 25.2, *p* < .001), ΔrCFI was higher than .01 (ΔrCFI = .106), and the AIC was lower for the 2-factor model.

**Fig 2:**
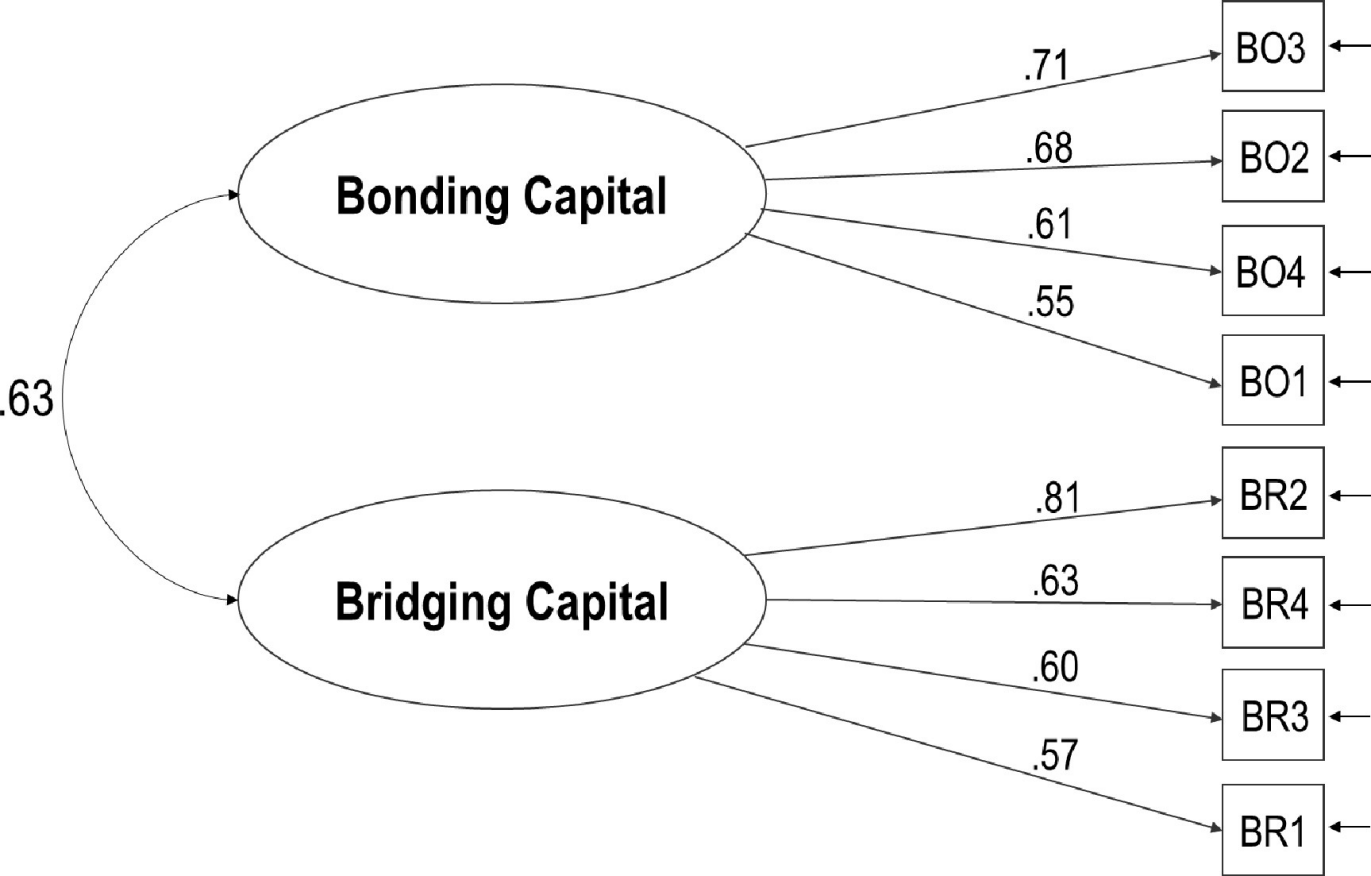
Confirmatory factor model of the 2-factor model of the personal on-offline social capital brief scale.

As shown in Fig 2, the factor loadings ranged from .55 to .81 (mean = .65, median = .62) and were all statistically significant. The interfactor correlation was moderate. Internal consistency was good for the total score (Cronbach’s alpha = .78; McDonald’s omega = .79) and for both bonding (Cronbach’s alpha = .71) and bridging (Cronbach’s alpha = .72) Social Capital dimensions. The statistical power of the model was excellent (.99).

### Discussion

The Personal On-Offline Social Capital Brief Scale showed the same 2-factor structure of bonding and bridging Social Capital found for both the long and brief versions of the PSCS. However, the explained variance greatly increased with the inclusion of items that measure online Social Capital. It seems that a relevant portion of an individual’s formal and informal networks and interactions are developed and maintained online. These results confirm that online social networks support bonding Social Capital [61] and that the use of the Internet is associated with bridging Social Capital [62]. It follows that a measurement of the online component of Social Capital must always be included in studies on Social Capital.

The Italian version of the Personal On-Offline Social Capital Brief Scale is available from the corresponding author under request. The Personal On-Offline Social Capital Brief Scale was translated into English according to the International Test Commission Guidelines for Translating and Adapting Tests [46]. The English version, verified by a professional Italian-English translator and a professor of psychology who is an American native English speaker, is available from the corresponding author under request. The contents investigated in the items are described in the Supporting information (S1 Appendix). The clearness and conciseness of items and instructions and the efficacy of the examples were investigated with field tests involving several American native English speakers.

## Third study: Convergent and divergent validity of the personal on-offline social capital brief scale

The third study aimed to investigate whether the bonding and bridging dimensions of the Personal On-Offline Social Capital Brief Scale enable the measurement of different traits. To this end, we verified its convergent and divergent validity in relation to scales measuring constructs with different patterns of relationships with bonding and bridging Social Capital. The Personal On-Offline Social Capital Brief Scale was administered with scales measuring the sense of community or the received social support from family, friends, or a special person, and health or stress. The sense of community refers to the perception of similarity and the feeling of being a member of a stable group, and the willingness to maintain interdependence with other members, giving to or doing for others what is expected of them [63]. Therefore, we hypothesized that (1.) bridging Social Capital (formal networks) is more closely related to the sense of community than bonding Social Capital (informal networks) and that (2.) bonding Social Capital is more closely related to perceived social support from friends than bridging Social Capital. Additionally, the Personal On-Offline Social Capital Brief Scale does not include resources associated with the family and one individual; accordingly, we hypothesized that (3.) both Social Capital dimensions are not associated with perceived support from family or a special person. Finally, as previous studies [64] suggested, we hypothesized (4.) absent or negative relationships of both Social Capital dimensions with health and stress.

### Materials and methods

#### Instruments

*Personal on-offline social capital brief scale*. See the second study.

*Italian Scale of Sense of Community (SISC)*. The sense of community developed in communities was measured with the SISC [65]. This scale comprises 18 items rated on a 4-point Likert scale that allows for the measurement of sense of belonging and emotional connection, needs and influences, social climate, and pleasantness of the environment. In the present investigation, internal consistency was good (Cronbach’s Alpha = .74).

*Multidimensional Scale of Perceived Social Support (MSPSS)*. Perceived social support was measured with the MSPSS [66, 67]. This scale consists of 12 items rated on a 6-point Likert scale organized into three subscales for the measurement of support and help received from family, friends, and a special person. In the present study, internal consistency was good (Cronbach’s alpha ranging from .86 to .95).

*Health survey Short-Form (SF-12)*. Physical and mental health was measured with the SF-12 Italian Version [68]. The scale comprises 12 items for the assessment of how the individual has been able to carry out the usual activities in the last four weeks. In the present investigation, the internal consistency was not fully sufficient (Cronbach’s alpha = .68).

*Perceived Stress Scale (PSS)*. Perceived stress was measured with the PSS [69]. This scale is composed of 10 items, each rated on a 5-point Likert scale, that allow measurement of the degree to which situations in a person’s life are evaluated as stressful. In the present study, the internal consistency was not fully sufficient (Cronbach’s alpha = .61).

*BIDR-6 short form*. See the first study.

#### Participants

Two independent groups, A and B, compiled two different booklets, A and B, respectively. Group A comprised 234 adults (30% men, mean age = 34.9 years [*SD* = 14.0], range = 18–72 years) living in Italian towns with different population sizes (less than 5 thousand individuals: 13%; 5–50 thousand individuals: 49%; 50–250 thousand individuals: 25%; and more than 250 thousand individuals: 13%). Among them, 4% had a third-year secondary school or a lower education level, 41% had a secondary school level education, and 55% had a postsecondary school or a higher educational level. The majority reported to be employed (74%). Among the 251 participants who had agreed to take part in the study, 17 were excluded because they exceeded the cutoff score for simulation on the BIDR-6 Short Form.

Group B included 247 adults (28% men, mean age = 36.6 years [*SD* = 14.9], range = 19–70 years) living in Italian towns with different population sizes (less than 5 thousand individuals: 19%; 5–50 thousand individuals: 44%; 50–250 thousand individuals: 26%; and more than 250 thousand individuals: 11%). Among them, 5% had a third-year secondary school or a lower education level, 36% had a secondary school level education, and 59% had a postsecondary school or a higher educational level. The majority reported to be employed (73%). Among the 267 participants who had agreed to take part in the study, 20 were excluded because they exceeded the cutoff score for social desirability responding on the BIDR-6 Short Form.

#### Procedure

Both booklets A and B were administered online. They included the Personal On-Offline Social Capital Brief Scale, the SISC and SF-12 (booklet A) or the MSPSS and PSS (booklet B), and the BIDR-6 Short Form. For each booklet, two versions were available to counterbalance the order of the SISC and SF-12 (SISC-SF-12 in 57% cases) or of MSPSS and PSS (MSPSS-PSS in 47% cases). The Personal On-Offline Social Capital Brief Scale and BIDR-6 were always in the first and last positions, respectively.

Data collection took place between November 2018 and June 2019. The online questionnaire was disseminated via services/institutes located in towns from northern to southern Italy, including cultural, community service, religious, political, and recreational groups/associations; protective services; shops; factories; and public and private schools. Participants took about 20 minutes to complete the survey.

This study was performed in accordance with the ethical standards laid down in the 2013 Fortaleza version of the Declaration of Helsinki. Written informed consent was obtained from each participant, and they did not receive any form of incentive. The Institutional Review Board Comitato Universitario di Bioetica, University of Perugia, approved the developed Personal On-Offline Social Capital Brief Scale (#2018-03R).

#### Data analysis

Four multiple regressions (one for Group A and three for Group B) were run for each bonding or bridging Personal On-Offline Social Capital Brief Scale dimension score as the criterion variable. Predictor variables were the scores on the SISC and SF-12 for Group A and the scores on the MSPSS family scale or friends scale or a special person scale and the PSS for Group B.

Regression assumptions were ascertained [70]: (1) the appropriateness of the number of participants was investigated in accordance with the assumption that *N ≥* 104 + number of predictors variables and calculating the statistical power of the regression with G*Power 3 [71]; (2) presence of univariate outliers (e.g., participants with *z* values higher than |3.29|) and multivariate outliers (e.g., participants for which the probability associated with the Mahalanobis distance was lower than .001) was checked for all the predictor and criterion variables. The normality of the univariate distribution was verified by computing asymmetry and kurtosis values, considered appropriate indices included in the range of −1.00 to 1.00. The normality of the multivariate distribution was verified using Mardia’s test; (3) multicollinearity among predictor variables was investigated by computing the tolerance index and the variance inflation factor (VIF), and the absence of collinearity was considered for values higher than .50 and lower than 2; (4) the normality, linearity and homoscedasticity of errors were verified by examining the shape of the residual distribution scatterplots and comparing the scatterplots of the obtained residual values with those of the theoretical values provided by Tabachnick and Fidell [70], both for the set of predictor variables and for each criterion variable; and (5) the independence of errors was investigated via Durbin-Watson statistics, considering appropriate values included in the range of 1.5–2.2, and the presence of outliers (e.g., extreme values higher than 3) was detected via the analysis of the standardized residuals.

### Results

The number of participants was appropriate and allowed to detect a small effect size (*f*^*2*^ = .04; α = .05, power = .80). Three univariate outliers were found in Group B and excluded (*n* = 244). No multivariate outliers were found, but the multivariate normality of the data distribution was not respected (the Mardia index’s value was slightly higher than the critical value). Multicollinearity and normality, linearity, homoscedasticity, and independence of errors were satisfied.

Among the two multiple regressions run in Group A, with SISC and SF-12 scores as predictor variables and the bonding or bridging Personal On-Offline Social Capital Brief Scale dimension score as the criterion variable, only the regression with bridging Social Capital dimension was significant (adjusted *R*^*2*^ = .03, *F*_(2,231)_ = 3.941, *p* = .021). SISC but not SF-12 resulted to positively associated with the bridging Social Capital dimension (*β* = .133, *p* = .05).

In Group B, six multiple regressions were run, three for each bonding and bridging Personal On-Offline Social Capital Brief Scale dimension as the criterion variable. The predictor variables were MSPSS family scale, or friends scale, or a special person scale, and PSS. The results were statistically significant for the two multiple regressions with the MSPSS friends scale and PSS as predictors of bonding (adjusted *R*^*2*^ = .05, *F*_(2,243)_ = 7.332, *p* = .001) and bridging (adjusted *R*^*2*^ = .03, *F*_(2,243)_ = 4.239, *p* = .016) Personal On-Offline Social Capital Brief Scale dimensions. The MSPSS friends scale was positively associated only with the bonding Social Capital dimension (*β* = .155, *p* = .014). In contrast, PSS was negatively associated with both bonding (*β* = -.161, *p* = .011) and bridging (*β* = -.178, *p* = .006) Social Capital dimensions.

### Discussion

The third study verified that bonding and bridging Social Capital dimensions of the Personal On-Offline Social Capital Brief Scale assess different aspects of Social Capital. The results showed that, in agreement with our first hypothesis, the sense of community resides in the relationships with the bridging Social Capital dimension, concerning formal networks with local and online groups and associations, but not with bonding Social Capital dimension, which concerns informal ties with friends, neighbors, and colleagues/fellow students. According to our second and third hypotheses, the support from friends is associated with the bonding but not bridging Social Capital dimension, whereas that from family or a special person is related neither to bonding nor to bridging Social Capital dimensions. It seems that having supportive friends leads to a greater bonding Social Capital, whereas a strong sense of belonging to the community leads to a wide bridging Social Capital.

Finally, in accordance with our fourth hypothesis, perceived health was not associated with bonding and bridging Social Capital dimensions, whereas perceived stress had a negative relationship with both dimensions. These results confirm that a higher level of stress is associated with a lower level of offline and online Social Capital [37, 72, 73]. However, reliability coefficients for the SF-12 and PSS were a bit low (.68 and .61, respectively); accordingly, the results should be interpreted carefully and need to be confirmed by further studies.

## General discussion

The Personal On-Offline Social Capital Brief Scale, a brief scale for the valid and reliable measurement of online and offline bonding and bridging Social Capital to be used in Italy, was developed by adapting the PSCS scale. It is composed of items organized in two dimensions that assess and discriminate the resources associated with informal networks (bonding Social Capital), and associations and groups (bridging Social Capital), and these networks are developed and maintained online to a large extent.

For the first time, this research identified the most relevant items of the PSCS for people living in an occidental country and included items to measure online Social Capital, which is becoming increasingly relevant [61, 62]. The developed Personal On-Offline Social Capital Brief Scale may be a useful instrument for obtaining information about all resources (e.g., support) associated with durable and trustworthy formal and informal networks that are crucial for planning public health interventions [8]. Indeed, studies have shown that Social Capital has positive effects on health outcomes [5, 10, 19]. Additionally, in the present study, it was found that a higher level of Social Capital is associated with a lower level of perceived stress.

A brief valid tool to measure Social Capital is also useful for the assessment of the sociocultural level. This is a multidimensional construct composed of the three dimensions of Socioeconomic Status (SES), Cultural Capital, and Social Capital (e.g., [60, 74]). Frequently, however, only SES is the object of assessment, as the sociocultural level is limited to the individual’s educational level, occupation, and income. Several investigations have found how SES, Cultural Capital, and Social Capital have different relationships with different aspects of human behavior. Consider, for example, the Big Five personality profiles of adolescents [43, 45], and adaptive behavior [42], and emotional and behavioral problems [44] of toddlers with autism spectrum disorders.

Further studies should investigate the invariance of the bonding and bridging factorial structure of the Personal On-Offline Social Capital Brief Scale for respondents differing in terms of educational level, age, nationality, or ethnic group. The stability of the model would thus also be confirmed. Finally, the Personal On-Offline Social Capital Brief Scale would also be useful for investigating any differences in Social Capital of individuals belonging to different subcultures (i.e., rural vs. urban; low SES vs. high SES).

## Conclusions

Overall, these studies provide evidence of reliability and validity related to the internal structure of the Personal On-Offline Social Capital Brief Scale and its relationships with other variables in measuring online and offline bonding and bridging SC and to discriminate them from similar constructs. This scale is a useful instrument for planning public health interventions.

## Supporting information

S1 Appendix Personal on-offline social capital brief scale. (DOCX)
