# Supplementary material for: Adaptation of the personal social capital brief scale for the measurement of the offline and online social capital in Italy
Source: PLoS One. 2022 Sep 1;17(9):e0272454. doi: 10.1371/journal.pone.0272454 (PMC9436046; doi:10.1371/journal.pone.0272454)
Supplement: S1 Appendix — (DOCX) [file pone.0272454.s001.docx]

**Supporting Information**

**Content Investigated in the Personal On-Offline Social Capital Brief Scale**

The individual is asked to read each question and check the box that best represents their answer on a 5-point Likert scale (A few/None-A lot/All).

| **1. Number of people in the individual’s social network for each of the following three categories** |
| --- |
| 1). Friends |
| 2). Work colleagues/fellow students |
| 3). Online contacts (i.e., persons met and with whom interact only online) |
| **2. Number of people in each of the following three categories who the individual can trust** |
| 1). Friends |
| 2). Work colleagues/fellow students |
| 3). Online contacts (i.e., persons met and with whom interact only online) |
| **3. Number of people in each of the following three categories who will definitely support the individual upon their request** |
| 1). Friends |
| 2). People in the neighborhood |
| 3). Online contacts (i.e., persons met and with whom interact only online) |
| **4. Number of people in all the following categories – individual’s friends,** **work colleagues/fellow students, people in the neighborhood, online contacts, and family members, relatives, fellow citizens or childhood friends/old classmates – who possess the following assets/resources** |
| 1). Broad connections with others |
| 2). High reputation/influence |
| **5. Number of the following two types of associations/groups in the individual’s community** |
| 1). Community service associations/groups (for example: groups, including youth organizations offering caregiving, assistance, shelter, soup kitchen, nursing home, environmental protection, trade union representation, cooperative associations, men’s groups, women’s groups, community committee/groups, local tourism promotion groups, student organizations) |
| 2). Cultural associations/groups including those having only online activities (for example: theatre or dance groups, music bands, choirs or orchestras, arts and crafts groups, traditional folk groups, promotional associations for cultural events, senior citizens’ study programs, youth-oriented cultural associations, Facebook/online discussion groups) |
| **6. Number of each of the two types of associations/groups in the individual’s community that represent their rights and interests** |
| 1). Community service associations/groups (for example: groups, including youth organizations offering caregiving, assistance, shelter, soup kitchen, nursing home, environmental protection, trade union representation, cooperative associations, men’s groups, women’s groups, community committee/groups, local tourism promotion groups, student organizations) |
| 2). Cultural associations/groups including having only online activities (for example: theatre or dance groups, music bands, choirs or orchestras, arts and crafts groups, traditional folk groups, promotional associations for cultural events, senior citizens’ study programs, youth-oriented cultural associations, Facebook/online discussion groups) |
| **7. Number of each of the two types of associations/groups in the individual’s community that will support them upon their request?** |
| 1). Community service associations/groups (for example: groups, including youth organizations offering caregiving, assistance, shelter, soup kitchen, nursing home, environmental protection, trade union representation, cooperative associations, men’s groups, women’s groups, community committee/groups, local tourism promotion groups, student organizations) |
| 2). Religious or political associations/groups |
| **8. Number of associations/groups in the individual’s community of the following categories– community service, cultural, religious, political, recreational, leisure, and economic and professional associations/groups (e.g., Chamber of Commerce) – that possess the following assets/resources** |
| 1). Broad social connections |
| 2). Extensive social influence |
